# Editorial: Mechanisms and Implications of the Aging of Cardiovascular Regenerative Cells

**DOI:** 10.3389/fcvm.2018.00093

**Published:** 2018-07-17

**Authors:** Antonio P. Beltrami, Gaia Spinetti

**Affiliations:** ^1^Department of Medicine, University of Udine, Udine, Italy; ^2^Laboratory of Cardiovascular Research, IRCCS MultiMedica, Milan, Italy

**Keywords:** aging, vascular, regeneration, IPS (induced pluripotent stem) cell, reactive oxygen species, cardiac

In this Research Topic, six groups discussed recent advances in the field of cardiovascular aging. In particular, the biology of vascular regenerative cells has been dissected. The topic is of particular relevance for scientists searching for solutions of the diseases of cardiovascular system, given the advancing age of the population of developed countries (1, 2). Articles cover many aspects of this problem, ranging from vascular aging, to cell reprogramming, touching organism development, senescence, and mechanisms responsible for the progressive loss of the reparative ability of tissue progenitors.

In their review, Moriya and Minamino discuss the topic of the aging of vascular cells and the consequent impairment in angiogenesis. This appears to be a double-edged sword condition: due to increased age-associated vascular cell senescence, old patients face delayed wound healing and less efficient response to ischemia, but they are also somehow protected from cancer that can progress only supported by new vascular structures that provide nutrition. Moreover, learning the mechanisms that regulate the cellular dysfunction with age is instrumental to improve the efficacy of therapeutic angiogenesis, a promising approach to cure ischemic diseases affecting mostly old people. Authors point at central factors that need to be finely tuned to boost angiogenesis without increasing malignancy. A central one is nitric oxide (NO), the production of which decreases in aged cells.

Since elderly are the group of patients who mostly would benefit from cell therapy, the question whether chronological age negatively impacts on induced pluripotent stem cell (iPSC) functionality is a hot topic. In the article by Strässler et al. several aspects of the potential problem represented by age-related limitations on the reprogramming process itself are addressed. Therefore, the expansion in culture, and the differentiation of old donor-derived iPSC are discussed. Somehow surprisingly, the reprogramming capabilities of somatic cells, although decreased with age, appears to be maintained as iPSC from centenarians indicate (3, 4). However, centenarians represent a group of subjects that may possess a genetic background that facilitate the escape from classical age-associated cell changes as we and others also demonstrated (5). Maintenance in culture and differentiation ability are re-started by the iPSC induction, but the obtained cellular product may still be damaged by karyotype aberrations and DNA mutations accumulated in the process. Thus, these limitations are still to be addressed before clinical exploitation may be possible.

The concept of cell senescence in cell reprogramming is discussed in the review by Passaro and Testa. Although the heart was considered to be a post-mitotic organ, it is now recognized that cardiac cells, including cardiomyocytes still possess the capability to proliferate and regenerate damages. Senescence can therefore be seen as a negative condition that slows down the replicative potential of cells, but it can also be beneficial for reprogramming by generating a pro-inflammatory environment by the acquisition of the senescent-associated secretory phenotype (SASP) (6). This novel concept is dissected in this article.

In their review, Castellan and Meloni discussed the transition between the regenerative postnatal phase and the maturity. Indeed, although neonatal mice can fully regenerate cardiac damage, this “regenerative window” is rapidly lost 1 week after birth. Although authors identified cardiomyocyte replenishment, regression of fibrosis, and neovascularization as the three key factors required for proper regeneration, most of the current effort is devoted to the identification of mechanisms unlocking cardiomyocyte renewal. In this regard, microRNAs (miR) have been intensively studied. Among these, authors discussed the role of miR-34a, members of the miR-15, miR-590, miR-199, and the miR-17-92 cluster in modulating adult cardiomyocyte proliferation. Authors subsequently discuss the role played by both the transcriptional co-activators YAP/TAZ and the transcription factor Meis1 in modulating the entry of cardiomyocytes into the cell cycle. Last, authors remark the role played by both immune cells (mostly macrophages) and that of multipotent, tissue resident stem cells in cardiac regeneration. Intriguingly, the importance of the latter in neonatal heart regeneration has not been assessed yet.

Angelini et al. on the other hand considered the role played by circulating factors on the physiology and senescence of cells that have been tested in cardiovascular regeneration trials. This is a hot topic, since heterochronic parabiosis experiments have clearly shown that blood-born factors can revert age-related tissue changes(7). The complex role of both Growth Hormone-GH-/IGF-1 signaling and GDF-11 in development and, most of all, aging is discussed. Next, authors present evidence of the involvement of PAI-1, a factor whose levels increase with organism aging, in senescence. Last, the role played by circulating miRNA in organism aging is described, focusing on miR-34, miR-146a, and miR-21.

Finally, Cesselli et al. reviewed the importance of redox signaling and oxidative stress in the pathophysiology of cardiovascular disease. Indeed, reactive oxygen species (ROS), whose production is strictly regulated from a time and spatial point of view, are neither good or bad in their essence, since they are important regulators of cell fate, including stem cell quiescence, self-renewal, and differentiation. Detrimental effects, such as oxidative stress occur when ROS production becomes less stringently controlled.

In conclusion, upon reaching maturity, mammalians lose the ability to robustly regenerate organ damage, with mechanisms, that are only partially understood. Moreover, complex, interconnected, age-dependent mechanisms, that include alteration of circulating factors, derangements of redox signaling and oxidative stress, and inflammation alter the biology of adult progenitors, negatively impacting the ability to repair tissue damages. However, cellular senescence ignites a paracrine response (SASP), which includes IL6, that is aimed at orchestrating tissue repair and may favor dedifferentiation and cell reprogramming in non-senescent cells. This relatively novel concept has important implications in the generation of iPSC from elderly people, that leaves a word of hope for those patients that will need the most from these novel approaches.

## Author contributions

GS and AB contributed equally to this article by generating ideas and writing the text.

### Conflict of interest statement

The authors declare that the research was conducted in the absence of any commercial or financial relationships that could be construed as a potential conflict of interest.
